# Diagnostic performance of the Sanity 2.0 assay to detect resistance to rifampicin, isoniazid, and fluoroquinolones in tuberculosis

**DOI:** 10.1128/jcm.01299-25

**Published:** 2025-12-10

**Authors:** Zhen Feng, Shijia Ge, Peiwen Miao, Mei Liu, Qing Li, Rong Li, Lingyun Song, Yilin Zhang, Feng Sun, Xinchang Chen, Yang Li, Wenhong Zhang

**Affiliations:** 1 Department of Infectious Diseases, Shanghai Key Laboratory of Infectious Diseases and Biosafety Emergency Response, National Medical Center for Infectious Diseases, Huashan Hospital, Shanghai Medical College, Fudan University 58305, Shanghai, China; 2 Department of infectious disease, Lanxi People’s Hospital, Jinhua, Zhejiang, China; 3 Department of Tuberculosis, Affiliated Hospital of Zunyi Medical University, Guizhou, China; 4 Shanghai Sci-Tech Inno Center for Infection & Immunity, Shanghai, China; The University of North Carolina at Chapel Hill School of Medicine, Chapel Hill, North Carolina, USA

**Keywords:** tuberculosis, the Sanity 2.0 assay, rapid molecular testing, drug resistance detection, drug susceptibility test

## Abstract

Effective tuberculosis (TB) management relies on prompt diagnosis of *Mycobacterium tuberculosis* complex (MTBC) and associated drug resistance. The Sanity 2.0 assay is a high-resolution melting assay designed for direct respiratory sample testing, enabling simultaneous detection of MTBC and resistance to rifampicin (RIF), isoniazid (INH), and fluoroquinolones (FQ) in a single step. This study evaluated its diagnostic performance in two registered multicenter trials among bacteriologically confirmed TB patients. Diagnostic performance was evaluated for MTBC detection, as well as for the identification of resistance to RIF, INH, and FQ, using phenotypic drug susceptibility testing, whole-genome sequencing, and a composite reference standard. Agreement analyses were conducted between the Sanity 2.0 assay and Xpert MTB/RIF and Xpert MTB/XDR. Among 611 patients, the Sanity 2.0 assay detected MTBC in 563 patients, exhibiting a sensitivity of 92.1% (95% CI: 89.7–94.0). For detecting resistance to RIF, INH, and FQ, sensitivities exceeded 90%, with specificities of 95.8% (95% CI: 88.5–98.6), 100.0% (95% CI: 96.4–100.0), and 97.8% (95% CI: 93.8–99.3) against the composite reference standard, respectively. The agreement with Xpert MTB/RIF for RIF detection was 98.6% (95% CI: 96.9–99.3). For INH and FQ resistance, the agreement with Xpert MTB/XDR was 92.0% (95% CI: 88.5–94.5) and 94.3% (95% CI: 91.2–96.3), respectively. The Sanity 2.0 assay is a rapid and user-friendly platform capable of detecting both MTBC and key drug resistance. It demonstrated good diagnostic performance and could potentially be an effective alternative to guide individualized anti-TB treatment, especially in resource-limited settings.

**IMPORTANCE:**

Rapid and accurate detection of both *Mycobacterium tuberculosis* complex (MTBC) and key drug resistance is critical to improving tuberculosis treatment outcomes and reducing transmission. However, current molecular diagnostic workflows often require sequential testing, which can delay the initiation of effective and individualized therapy. We evaluated the Sanity 2.0 assay, an integrated high-resolution melting test that simultaneously detects MTBC and resistance to rifampicin, isoniazid, and fluoroquinolone resistance directly from respiratory samples in about 2–3 hours. The assay demonstrated excellent performance, with MTBC detection sensitivity of 92.1% and drug resistance sensitivities exceeding 90% and specificities over 95% against a composite reference standard, as well as strong concordance with World Health Organization-endorsed molecular assays. Implementation of the Sanity 2.0 assay could streamline TB diagnostic workflows; enable rapid, single-step resistance profiling; and facilitate timely, individualized treatment—particularly in resource-limited settings where rapid and comprehensive resistance testing remains a critical unmet need.

## INTRODUCTION

Tuberculosis (TB) remains a major global public health threat (1). In 2023, an estimated 10.8 million people developed TB, including 400,000 cases of multidrug-resistant or rifampicin-resistant TB (MDR/RR-TB), but only 8.2 million and 189,000 cases were diagnosed, respectively (1). One of the key challenges of TB control is MDR/RR-TB, with only about one-third of patients receiving treatment and success rates remaining suboptimal (1). Delays in diagnosis and treatment initiation, along with the complexity of drug-resistant TB (DR-TB) regimens, contribute substantially to poor outcomes. To address these challenges, the World Health Organization (WHO) recommends rapid molecular diagnostics as the initial diagnostic tool for individuals with suspected TB (2). Rapid and accurate diagnosis of both *Mycobacterium tuberculosis* complex (MTBC) and drug resistance would increase case detection and ensure timely initiation of effective therapy (3), thereby reducing transmission, morbidity, and mortality (4, 5).

Fluoroquinolones (FQ) are pivotal in the treatment of all major forms of tuberculosis—including drug-susceptible TB (DS-TB), DR-TB, and isoniazid-resistant, rifampicin-susceptible TB (Hr-TB). According to the WHO recommendations, a shorter 4-month moxifloxacin-based regimen is available as an alternative to the conventional 6-month regimen for DS-TB, whereas a 6-month regimen consisting of bedaquiline, pretomanid, linezolid, and moxifloxacin (BPaLM) is endorsed for MDR/RR-TB (6). FQs are also essential for treating Hr-TB, which is more prevalent than MDR/RR-TB and associated with poorer treatment outcomes (7). Nevertheless, isoniazid (INH) resistance often goes undetected due to limited accessibility of reliable drug susceptibility testing (DST) (4).

Accurate and timely detection of resistance to rifampicin (RIF), INH, and FQ before treatment initiation is therefore critical for appropriate regimen selection and improved outcomes (6). Current WHO-recommended diagnostic workflows rely on a stepwise approach—initially detecting MTBC and rifampicin resistance, followed by additional assays to assess FQ and INH resistance. This multi-step process might impose time and cost burdens and greater procedural complexity, especially in resource-limited settings.

These challenges underscore the urgent need for rapid, accurate, and cost-effective molecular diagnostics that can simultaneously detect MTBC and resistance to RIF, INH, and FQs in a single test. The Sanity 2.0 assay (Zeesan Biotech, Xiamen, China), a high-resolution melting assay, is an innovative molecular test designed to address this gap. The assay integrates nucleic acid extraction and amplification within a cartridge and employs fluorescence polymerase chain reaction (PCR) melting curve analysis to identify resistance-associated mutations directly from respiratory specimens. By combining automated operation with rapid turnaround time, it is designed to meet the urgent need for timely and reliable drug resistance testing.

This study aimed to evaluate the diagnostic performance of the Sanity 2.0 assay for the detection of MTBC and resistance to RIF, INH, and FQ using phenotypic drug susceptibility testing (pDST), whole-genome sequencing (WGS), and the composite reference standard as reference. The secondary objective was to assess the assay’s ability to detect resistance to RIF, INH, and FQ in comparison to Xpert MTB/RIF and Xpert MTB/XDR. In addition, the study investigated the possible reasons for the discrepancies between the Sanity 2.0 assay and WGS or pDST results.

## MATERIALS AND METHODS

### Study participants and procedures

Participants were enrolled from two registered trials in China (ORIENT: NCT 5401071, INSPIRE: NCT 5081401) between 1 April 2022 and 30 March 2024. The two trials aimed to optimize regimens for DS-TB and MDR/RR-TB, respectively. In ORIENT (8), participants were eligible if their respiratory specimens (including sputum and bronchoalveolar lavage fluid [BALF]) tested positive either for acid-fast bacilli by smear microscopy or for MTBC by the Xpert MTB/RIF assay (Cepheid, Sunnyvale, CA, USA). In INSPIRE, eligible participants were RR-TB patients confirmed by molecular DST (including Xpert MTB/RIF, Cepheid, Sunnyvale, CA, USA; GenoTypeMTBDRplus VER2.0, Hain Lifescience GmbH, Nehren, Germany; MeltPro MTB/RIF Test Kit, Zeesan Biotech, Xiamen, China; DNA microarray chip method, CapitalBio., Beijing, China) or by phenotypic DST (as described in the pDST section).

Eligible patients from both trials were included in the current study if sufficient respiratory samples were available for the Sanity 2.0 testing. When additional specimens were available after Sanity 2.0 testing, the residual samples were subsequently tested by Xpert MTB/XDR. WGS and/or pDST were performed on all culture-positive isolates.

Information on patients’ sociodemographic characteristics, radiological findings, treatment history, and drug resistance profiles was systematically collected. The study was approved by the ethics committees of Huashan Hospital and all participating sites, and written informed consents were obtained from all participants.

### Sanity 2.0 TB assay

Prior to sample loading, sputum specimens were mixed with a 2-fold volume of treatment solution (provided in the kit, mainly containing sodium hydroxide), whereas BALF samples were mixed with 0.5 times the volume of the solution, respectively, in sterile containers. Following 30 seconds of vortex mixing, the mixtures were incubated at room temperature for 10 min to ensure complete liquefaction. Upon completion of liquefaction, the Sanity 2.0 assay was performed according to the manufacturer’s protocol. Briefly, 1 mL of the digested respiratory specimen was transferred to the extraction loading well of the extraction cartridge. Both cartridges (extraction and test) were then securely positioned in their designated modules, and the running program was pre-matched by scanning the QR codes located on the cartridges. Clicking the "Run" button triggered the automated testing process. After running, the instrument automatically outputs the results for MTBC detection and resistance to RIF, INH, and FQ.

Within the instrument, nucleic acids were extracted and subjected to multiplex real-time PCR amplification. Fluorescent probes specifically hybridized to resistance-associated regions of the *rpoB*, *katG*, *inhA*, *ahpC*, and *gyrA* genes. Following amplification, the system performed high-resolution melting analysis, where differences in melting temperature (Tm) from the wild-type control curves were used to identify nucleotide substitutions indicative of resistance. The total turnaround time from sample loading to the final result output was approximately 2 h.

### WGS and PDST

At local site laboratories, respiratory specimens were processed using the standard N-acetyl-L-cysteine–NaOH method and cultured on Löwenstein-Jensen solid media or in BACTEC Mycobacterial Growth Indicator Tube (MGIT; Becton Dickinson and Co., Franklin Lakes, NJ, USA) liquid media with the MGIT 960 system. All positive cultures were confirmed as MTBC by MPT64 antigen (Hangzhou Innovation Biotechnology, Hangzhou, China) detection. *Mycobacterium tuberculosis* isolates underwent DST at the site laboratories. Phenotypic DST was performed using the broth microdilution plate, MGIT, or Löwenstein-Jensen method, adhering to the 2018 updated WHO recommended critical concentrations and the 2022 updated WHO optimized broth microdilution plate methodology (9, 10).

WGS was performed on all culture-positive isolates. Genomic DNA was extracted using a Total Nucleic Acid Extraction Kit (IngeniGen XMK Biotechnologies, Inc., Zhejiang, China), and sequencing libraries were constructed using an IngeniGen DNA Library Preparation Kit (IngeniGen XMK Biotechnology), following the manufacturer’s protocol. Sequencing was performed on the NovaSeq 6000 system with a minimum 100-fold coverage. The WGS data were analyzed with reference to previous studies published by the author (11–13). After filtering the low-quality reads, the reads were aligned to the MTB H37Rv (GenBank NC000962.3) reference sequence using Bowtie2 (version 2.3.3.1) with default parameters. Single-nucleotide polymorphisms (SNPs) were detected using SAMtools (version 1.6) with a minimum sequencing depth of 10 reads without strand bias. All SNPs with a mutation frequency exceeding 10% were retained, enabling the detection of minor subpopulations and microevolution. The mutations related to resistance were according to the WHO mutation catalog (14).

### Performance analysis

Diagnostic performance of the Sanity 2.0 TB assay was evaluated using the following outcome measures: (i) the sensitivity for MTBC detection among bacteriologically confirmed TB cases, defined as patients with positive results for MTBC by either Xpert MTB/RIF or culture (6). (ii) The sensitivity, specificity, positive predictive value (PPV), negative predictive value (NPV), and overall accuracy rate for detecting resistance to RIF, INH, and FQ, using pDST and WGS independently and combined as reference standards. The composite reference results were classified as “Resistant” if either pDST or WGS indicated “Resistant” and “Susceptible” only if both pDST and WGS were “Susceptible.” (iii) The positive percentage agreement (PPA), negative percentage agreement (NPA), and agreement rate for the resistance detection of RIF, INH, and FQ compared with Xpert MTB/RIF and Xpert MTB/XDR. (ⅳ) The proportion of “indeterminate” results obtained via the Sanity 2.0 assay was evaluated. Indeterminate test results were excluded from the drug resistance detection performance analysis.

Statistical analysis was conducted using R (version 4.3.1), and the 95% confidence intervals (95% CI) for sensitivity, specificity, PPV, and NPV were calculated using the Wilson score method. Comparisons between categorical variables were performed using the χ^2^ or Fisher exact tests, and continuous variables were compared using the Mann–Whitney U test. *P* values of < 0.05 were considered statistically significant.

## RESULTS

### Patients’ characteristics

**Fig 1:**
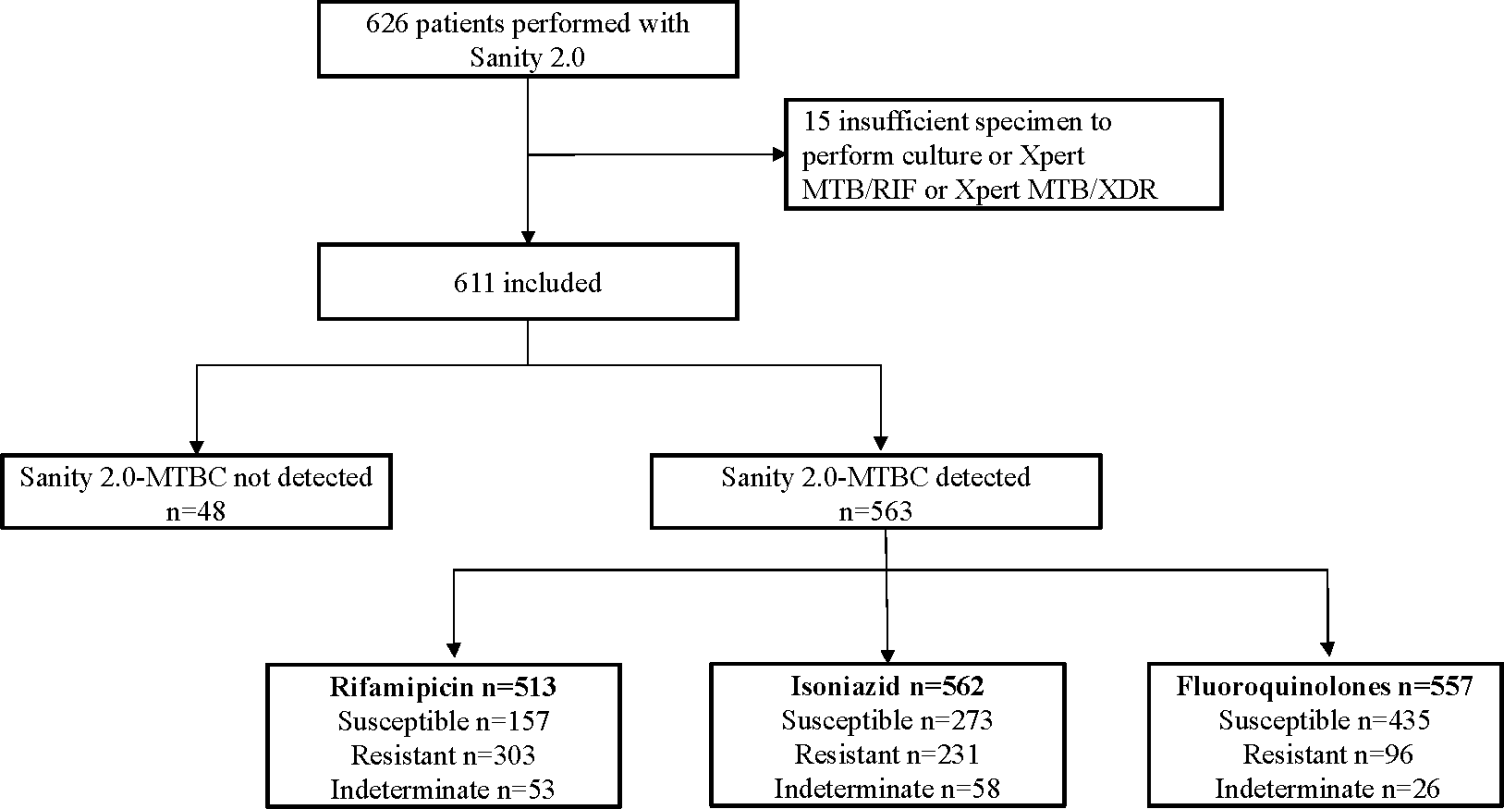
Participant enrollment and exclusions. Abbreviation: MTBC, *Mycobacterium tuberculosis* complex.

Between 1 April 2022 and 30 March 2024, the Sanity 2.0 assay was performed in 507 sputum and 119 BALF samples from a total of 626 patients. Fifteen were excluded due to insufficient specimen volume for reference testing (culture, Xpert MTB/RIF, or Xpert MTB/XDR) (Fig. 1). Consequently, 611 patients were included in the final analysis, of whom 69% were male, with a median age of 41 years. Among the 611 patients, 255 (41.7%) were enrolled from the ORIENT trial, and 356 (58.3%) from the INSPIRE trial. More than half of the enrolled patients were new TB cases (364 [59.6%]). The smear-positive percentage was 54.7%. Additional sociodemographic and clinical characteristics are summarized in Table 1.

**TABLE 1:** Baseline characteristics of the patients^*a*^

Variable	Overall (*n* = 611)	INSPIRE (DR-TB cohort) (*n* = 356)	ORIENT (DS-TB cohort) (*n* = 255)
Age, median (IQR)	41.0 (29.0, 53.0)	46.0 (33.0,55.0)	34.0 (25.0,50.5)
Sex, n (%)			
Male	423 (69.2%)	259 (72.8%)	164 (64.3%)
Female	185 (30.3%)	94 (26.4%)	91 (35.7%)
Unknown	3 (0.5%)	3 (0.84%)	0 (0.00%)
Body mass index, median (IQR)	20.0 (18.3, 22.2)	20.0 (18.0,22.2)	20.3 (18.4,22.2)
Smoking history, n (%)			
Yes	188 (30.8%)	116 (32.6%)	72 (28.2%)
No	381 (62.4%)	198 (55.6%)	183 (71.8%)
Unknown	42 (6.9%)	42 (11.8%)	0 (0.00%)
Previous history of tuberculosis, n (%)			
Yes	205 (33.6%)	171 (48.0%)	34 (13.3%)
No	364 (59.6%)	143 (40.2%)	221 (86.7%)
Unknown	42 (6.9%)	42 (11.8%)	0 (0.00%)
Presence of cavity, n (%)			
Yes	388 (63.5%)	263 (73.9%)	172 (67.5%)
No	181 (29.6%)	51 (14.3%)	83 (32.5%)
Unknown	42 (6.9%)	42 (11.8%)	0 (0.00%)
Bilateral involvement, n (%)			
Yes	435 (71.2%)	263 (73.9%)	172 (67.5%)
No	134 (21.9%)	51 (14.3%)	83 (32.5%)
Unknown	42 (6.9%)	42 (11.8%)	0 (0.00%)
Smear^*b*^, n (%)			
Positive	334 (54.7%)	230 (64.6%)	104 (40.8%)
Negative	229 (37.5%)	83 (23.3%)	146 (57.3%)
Unknown	48 (7.9%)	43 (12.1%)	5 (1.96%)
Sample type, n (%)			
Sputum	494 (80.9%)	296 (83.1%)	198 (77.6%)
Bronchoalveolar lavage fluid	117 (19.1%)	60 (16.9%)	57 (22.4%)

^*a*^ IQR, interquartile range; DS-TB, drug-susceptible tuberculosis; DR-TB, drug-resistant tuberculosis.

^*b*^ Percentages may not sum to 100% due to rounding.

### Diagnostic performance of Sanity 2.0 assay for the detection of MTBC

As shown in Fig. 1 and Table 2, the Sanity 2.0 assay detected MTBC in 563 patients, yielding an overall sensitivity of 92.1% (95% CI: 89.7–94.0). A total of 48 bacteriologically confirmed TB cases were not detected by the Sanity 2.0 assay. Comparative analysis (Table S1) showed that assay-negative cases had significantly lower body mass index (BMI), fewer cavitary lesions, lower smear positivity, and lower culture positivity than assay-positive cases, consistent with a paucibacillary disease profile. There were no significant differences in age, sex, or previous TB history between the two groups.

**TABLE 2:** Diagnostic performance of the Sanity 2.0 assay for the detection of MTBC in bacteriologically confirmed TB cases^*a*^

Test	N	TP	FP	FN	TN	Sensitivity (% [95% CI])
Sanity 2.0	611	563	0	48	0	92.1 (89.7–94.0)

^*a*^ TB, tuberculosis; MTBC, *Mycobacterium tuberculosis* complex; TP, true positive; FP, false positive; FN, false negative; TN, true negative; CI, confidence intervals.

Among the 563 MTBC-positive patients, 513 were tested for RIF resistance, with 303 (59.1%) identified as RIF-resistant, 157 (30.6%) as RIF-susceptible, and 53 (10.3%) yielding indeterminate results. For INH resistance, 562 patients were tested: 231 (41.1%) were INH-resistant, 273 (48.6%) were INH-susceptible, and 58 (10.3%) were indeterminate. For FQ resistance, 557 patients were tested: 96 (17.2%) were FQ-resistant, 435 (78.1%) were FQ-susceptible, and 26 (4.7%) were indeterminate.

### Diagnostic performance of the Sanity 2.0 assay for drug resistance

The diagnostic performance of the Sanity 2.0 assay for detecting RIF, INH, and FQ resistance was evaluated against pDST, WGS, and a composite reference standard, as summarized in Tables 3 to 5. Further performance details stratified by sample type (sputum vs. BALF) are shown in Table S2.

**TABLE 3:** Diagnostic performance of the Sanity 2.0 assay for detection of drug resistance to RIF compared with phenotypic drug susceptibility testing, whole-genome sequencing assay, and composite reference standard^*a*^

Drug	Reference standard	N	TP	FP	FN	TN	Sensitivity (% [95% CI])	Specificity (% [95% CI])	Accuracy (% [95% CI])	PPV (% [95% CI])	NPV (% [95% CI])
RIF	pDST	264	132	19	0	113	100.0 (97.2–100.0)	85.6 (78.6–90.6)	92.8 (89.0–95.3)	87.4 (81.2–91.8)	100.0 (96.7–100.0)
RIF	WGS	304	210	5	1	88	99.5 (97.4–99.9)	94.6 (88.0–97.7)	98.0 (95.8–99.1)	97.7 (94.7–99.0)	98.9 (93.9–99.8)
RIF	Composite reference	316	243	3	1	69	99.6 (97.7–99.9)	95.8 (88.5–98.6)	98.7 (96.8–99.5)	98.8 (96.5–99.6)	98.6 (92.3–99.7)

^*a*^ RIF, rifampicin; pDST, phenotypic drug susceptibility testing; WGS, whole-genome sequencing; TP, true positive; FP, false positive; FN, false negative; TN, true negative; PPV, positive predictive value; NPV, negative predictive value; CI, confidence intervals.

**TABLE 4:** Diagnostic performance of the Sanity 2.0 assay for the detection of drug resistance to INH compared with phenotypic drug susceptibility testing, whole-genome sequencing assay, and composite reference standard^*a*^

Drug	Reference standard	N	TP	FP	FN	TN	Sensitivity (% [95% CI])	Specificity (% [95% CI])	Accuracy (% [95% CI])	PPV (% [95% CI])	NPV (% [95% CI])
INH	pDST	296	111	6	10	169	91.7 (85.5–95.4)	96.6 (92.7–98.4)	94.6 (91.4–96.6)	94.9 (89.3–97.6)	94.4 (90.0–96.9)
INH	WGS	323	160	6	4	153	97.6 (93.9–99.0)	96.2 (92.0–98.3)	96.9 (94.4–98.3)	96.4 (92.3–98.3)	97.5 (93.6–99.0)
INH	Composite reference	303	189	0	12	102	94.0 (89.9–96.6)	100.0 (96.4–100.0)	96.0 (93.2–97.7)	100.0 (98.0–100.0)	89.5 (82.5–93.9)

^*a*^ INH, isoniazid; pDST, phenotypic drug susceptibility testing; WGS, whole-genome sequencing; TP, true positive; FP, false positive; FN, false negative; TN, true negative; PPV, positive predictive value; NPV, negative predictive value; CI, confidence intervals.

**TABLE 5:** Diagnostic performance of the Sanity 2.0 assay for the detection of drug resistance to FQ compared with phenotypic drug susceptibility testing, whole-genome sequencing assay, and composite reference standard^*a*^

Drug	Reference standard	N	TP	FP	FN	TN	Sensitivity (% [95% CI])	Specificity (% [95% CI])	Accuracy (% [95% CI])	PPV (% [95% CI])	NPV (% [95% CI])
FQ	pDST	228	27	11	3	187	90.0 (74.4–96.5)	94.4 (90.3–96.9)	93.9 (90.0–96.3)	71.1 (55.2–83.0)	98.4 (95.5–99.5)
FQ	WGS	335	60	8	3	264	95.2 (86.9–98.4)	97.1 (94.3–98.5)	96.7 (94.2–98.2)	88.2 (78.5–93.9)	98.9 (96.7–99.6)
FQ	Composite reference	212	69	3	6	134	92.0 (83.6–96.3)	97.8 (93.8–99.3)	95.8 (92.1–97.8)	95.8 (88.5–98.6)	95.7 (91.0–98.0)

^*a*^ FQ, fluoroquinolones; pDST, phenotypic drug susceptibility testing; WGS, whole-genome sequencing; TP, true positive; FP, false positive; FN, false negative; TN, true negative; PPV, positive predictive value; NPV, negative predictive value; CI, confidence intervals.

#### Rifampicin

A total of 264 patients had available results for both the Sanity 2.0 assay and pDST, and the assay showed a sensitivity of 100.0% (95% CI: 97.2–100.0) and a specificity of 85.6% (95% CI: 78.6–90.6). Among 304 patients compared with WGS, sensitivity and specificity were 99.5% (95% CI: 97.4–99.9) and 94.6% (95% CI: 88.0–97.7), respectively. When the composite reference standard was used (*n* = 316), the assay yielded a sensitivity of 99.6% (95% CI: 97.7–99.9) and specificity of 95.8% (95% CI: 88.5–98.6) (Table 3). When stratified by specimen type and compared with the composite reference standard, sensitivity for RIF resistance detection reached 99.5% in sputum and 100.0% in BALF, with specificities of 97.0% and 83.3%, respectively (Table S2).

#### Isoniazid

For INH resistance detection, 296 patients had available results for both the Sanity 2.0 assay and pDST, yielding a sensitivity of 91.7% (95% CI: 85.5–95.5) and a specificity of 96.0% (95% CI: 92.0–98.0). In the 324 patients with available WGS results, the assay demonstrated a sensitivity of 97.6% (95% CI: 93.9–99.0) and a specificity of 96.2% (95% CI: 92.0–98.3). Using the composite reference standard across 303 samples, the sensitivity and specificity were 94.0% (95% CI: 89.9–96.6) and 100.0% (95% CI: 96.4–100.0), respectively (Table 4). Compared with the composite reference standard, the assay maintained high diagnostic performance across both sputum and BALF samples. The assay achieved a sensitivity of 93.4% in sputum and 97.1% in BALF, both with 100.0% specificity (Table S2).

We further analyzed the characteristics of discrepant cases with a high number of false negatives for INH resistance compared with the composite reference standard. As shown in Table S3, false-negative and true-positive cases exhibited similar demographic and clinical characteristics. All false-negative patients had a previous history of tuberculosis (100% vs. 51.1%, *P* = 0.002).

#### Fluoroquinolones*** ***

The results for the Sanity 2.0 assay and pDST for FQ were available for 228 patients. Using pDST as the reference, the Sanity 2.0 assay showed a sensitivity of 90.0% (95% CI: 74.4–96.5) and specificity of 94.4% (95% CI: 90.3–96.9). The diagnostic performance was better using WGS as the reference (*n* = 335), sensitivity was 95.2% (95% CI: 86.9–98.7), and specificity reached 97.0% (95% CI: 94.3–98.5). Among 212 patients compared with the composite reference standard, sensitivity was 92.0% (95% CI: 83.6–96.3), and specificity was 97.8% (95% CI: 93.8–99.3) (Table 5). In the stratified analysis based on the composite reference standard, sensitivities reached 90.3% in sputum and 100.0% in BALF, with corresponding specificities of 99.2% and 88.2%, respectively (Table S2).

#### Agreement with the established molecular diagnostic Xpert

Consistencies between the Sanity 2.0 assay and comparator molecular diagnostics (Xpert MTB/RIF and Xpert MTB/XDR) were assessed in detecting drug resistance. For RIF resistance, the Sanity 2.0 assay demonstrated a PPA of 98.7% (95% CI: 96.6–99.5) and a NPA of 98.3% (95% CI: 93.9–99.5) compared with Xpert MTB/RIF. For INH, 26 discrepant results were identified in comparison to Xpert MTB/XDR. The PPA was 90.9% (95% CI: 86.5–94.0), whereas the NPA reached 95.2% (95% CI: 88.3–98.1).

For FQ, compared with the Xpert MTB/XDR, the Sanity 2.0 assay showed a PPA of 88.0% (95% CI: 79.8–93.2) and a NPA of 96.7% (95% CI: 93.6–98.3) (Table 6). Further analysis of discrepant cases with low PPA for FQ resistance (Table S4) revealed no significant demographic or clinical differences between Sanity-S/Xpert-R and Sanity-R/Xpert-R cases.

**TABLE 6:** Diagnostic performance of the Sanity 2.0 assay for detection of drug resistance compared with Xpert MTB/RIF and Xpert MTB/XDR assays^*a*^

Drug	Reference standard	N	No. of Sanity-R/ Xpert-R	No. of Sanity-R/ Xpert-S	No. of Sanity-S/ Xpert-R	No. of Sanity-S/ Xpert-S	PPA (% [95% CI])	NPA (% [95% CI])	Agreement (% [95% CI])
RIF	Xpert MTB/RIF	415	295	2	4	114	98.7 (96.6–99.5)	98.3 (93.9–99.5)	98.6 (96.9–99.3)
INH	Xpert MTB/XDR	314	210	4	21	79	90.9 (86.5–94.0)	95.2 (88.3–98.1)	92.0 (88.5–94.5)
FQ	Xpert MTB/XDR	332	81	8	11	232	88.0 (79.8–93.2)	96.7 (93.6–98.3)	94.3 (91.2–96.3)

^*a*^ RIF, rifampicin; INH, isoniazid; FQ, fluoroquinolones; R, drug-resistant; S, drug- susceptible; PPA, positive percent agreement; NPA, negative percent agreement; CI, confidence interval.

### Discrepant result resolution

#### Rifampicin

Among the patients tested for RIF resistance, 22 showed discrepant results between the Sanity 2.0 assay and pDST and/or WGS (Table S5). Of these, 16 patients (72.7%) were identified as RIF-resistant by the Sanity 2.0 assay, but only discordant with pDST. Among them, 13 had molecular evidence of resistance confirmed by both WGS and Xpert MTB/RIF, whereas the remaining three patients were consistent with Xpert MTB/RIF but lacked WGS data.

Four patients (18.2%) showed discordance between the Sanity 2.0 assay and WGS. Notably, one patient was reported as RIF-susceptible by the Sanity 2.0 assay but harbored a *rpoB*_V170F mutation located outside the assay’s target region. The other three patients were identified as RIF-resistant by the Sanity 2.0 assay but susceptible by WGS; among them, one was also susceptible by pDST, and all three were consistent with Xpert MTB/RIF.

In two additional patients (9.1%), the Sanity 2.0 assay indicated RIF resistance, whereas all reference methods (pDST, WGS, and Xpert MTB/RIF) reported susceptibility, suggesting potential false-positive results.

#### Isoniazid

For INH resistance, 24 patients exhibited discrepant results compared with pDST and/or WGS (Table S6). Of these, 14 (58.3%) showed inconsistency with pDST results; eleven of these cases were concordant with WGS, whereas three patients lacked WGS data, but one was confirmed by Xpert MTB/XDR.

Among eight patients (33.3%) with discordance between the Sanity 2.0 assay and WGS, five were identified as INH-resistant by the Sanity 2.0 assay and confirmed by either pDST or Xpert MTB/XDR. The remaining patient demonstrated inconsistent results with both WGS and Xpert MTB/XDR. Two patients missed by the Sanity 2.0 assay but found to harbor the *katG*_152A_deletion and *katG*_W198* mutations outside the assay’s detected region.

Additionally, two (8.3%) patients were classified as susceptible by the Sanity 2.0 assay but resistant by both pDST and WGS and were concordant with Xpert MTB/XDR.

#### Fluoroquinolones

Regarding FQ resistance, 22 discrepant results were identified in comparison with pDST and/or WGS (Table S7). Eleven (50%) patients showed inconsistency with pDST, of whom seven patients disagreed with pDST but aligned with WGS, and one patient matched Xpert MTB/XDR.

Among nine patients (40.9%) with discordance between the Sanity 2.0 assay and WGS, eight were concordant with Xpert MTB/XDR. One patient reported as susceptible by the Sanity 2.0 assay harbored a *gyrB*_D461N mutation undetectable by the assay. In two cases, the Sanity 2.0 assay identified resistance, but WGS interpreted *gyrA*_D94V and *gyrA*_D89N as susceptible.

Finally, two patients (9.1%) exhibited inconsistent results across all methods, suggesting a possible false-positive result by the Sanity 2.0 assay.

## DISCUSSION

In this multicenter clinical study, the Sanity 2.0 assay was evaluated for the first time and demonstrated satisfactory diagnostic performance in simultaneously detecting MTBC and resistance to key anti-TB drugs, including RIF, INH, and FQ. The detection performance was comparable with that of WHO-endorsed molecular assays. Although treatment paradigms for DR-TB and DS-TB have continued to evolve (6), the Sanity 2.0 assay remains a promising tool for identifying and guiding the treatment of DS-TB, MDR/RR-TB, isoniazid-resistant, fluoroquinolone-resistant, and pre-extensively drug-resistant tuberculosis (pre-XDR-TB).

The Sanity 2.0 assay met the WHO-recommended performance thresholds for low-complexity molecular diagnostics, demonstrating a sensitivity of 92.1% for MTBC detection—exceeding the minimum criteria of ≥90% sensitivity outlined in the WHO guidance (4). The diagnostic performance was comparable with that of previously reported assays, including Xpert Ultra (pooled sensitivity 90.9%) and Xpert MTB/RIF (pooled sensitivity 84.7%) (15, 16). This high sensitivity supports its potential utility as a screening tool. False-negative results were not retested in this study due to limited specimen availability; however, further investigations, including repeat testing where sufficient specimens are available, may help refine performance estimates of the Sanity 2.0 assay for MTBC detection.

The clinical performance of the Sanity 2.0 assay in this study was comparable with that of other WHO-endorsed molecular diagnostic platforms (17–19), with sensitivities exceeding 90% for RIF, INH, and FQ when compared with pDST. Furthermore, the assay met the minimum sensitivity thresholds defined in the WHO Target Product Profile for next-generation DST assays (4)—specifically, >95% sensitivity for RIF and >90% for INH and FQ. When considering WGS alone as the reference standard, the clinical performance of the Sanity 2.0 assay for all three drug targets increased to >95% sensitivity and >94% specificity. However, the specificity for RIF was relatively low when using pDST as the reference, at 85.6% (95% CI: 78.6–90.6). When WGS was used as the reference standard, the specificity improved to 94.6% (95% CI: 88.0–97.7). Compared with Xpert MTB/RIF, the Sanity 2.0 assay achieved a high PPA of 98.7% (95% CI: 96.6–99.5). The strong agreement between molecular assays suggests that the discrepancies with pDST may be due to fundamental discordances between genotypic and phenotypic methods. Molecular assays detect genetic mutations known to confer resistance, whereas pDST reflects actual inhibition of mycobacterial growth in culture, and the two approaches may not always correlate—particularly in cases involving disputed mutations or borderline resistance. These discrepancies underscore the importance of integrating molecular results with clinical and epidemiological context when interpreting drug resistance.

The performance of the Sanity 2.0 assay was also evaluated in sputum and BALF, which demonstrated highly reliable performance in sputum, maintaining high sensitivity (≥90%) and specificity (≥97%), indicating reliable performance under routine diagnostic conditions. Although the overall diagnostic accuracy of BALF remained consistent, the 95% confidence intervals for key parameters (sensitivity and specificity) were wider, primarily due to the relatively small sample size of the BALF cohort (*N* = 30–47) rather than fundamental differences in the assays.

An important advantage of the Sanity 2.0 assay is its ability to simultaneously detect drug resistance to RIF, INH, and FQ directly from respiratory specimens, enabling timely clinical decision-making. Given the substantial variability in drug resistance profiles across regions (20, 21) and the central role of FQ in both MDR/RR-TB and DS-TB regimens (22–25), early identification of FQ resistance is crucial. The fifth national TB epidemiological survey of China in 2010 reported that 7.4% of new TB patients harbored moxifloxacin-resistant strains (26, 27). In high TB burden countries, FQ is often empirically prescribed for other conditions before TB is diagnosed, further fueling resistance (28, 29). Moreover, delays in treatment initiation due to the long turnaround time of phenotypic DST and frequent treatment interruptions due to adverse events in DR-TB necessitate rapid molecular DST tools (30). In this study, the Sanity 2.0 assay, evaluated against WHO-endorsed molecular diagnostics and a composite reference standard, showed high diagnostic accuracy for all three drugs, supporting its utility in guiding clinical regimen selection, including in patients requiring salvage regimens.

Beyond its diagnostic accuracy, the Sanity 2.0 assay offers notable practical advantages. It supports flexible throughput (1–4 samples per run) and is based on fluorescence PCR melting curve analysis performed within a cartridge. The assay integrates nucleic acid extraction and amplification into a single streamlined workflow, greatly reducing manual handling and sample transfer time. The total time for the Sanity 2.0 assay is about 140–200 min, including 13–15 min of hands-on operation, which is comparable with the Xpert MTB/RIF (140–150 min, 20–30 min hands-on). Moreover, its compact and self-contained design allows for flexible deployment across varied laboratory environments, including those with limited infrastructure. These features make the Sanity 2.0 assay particularly suitable for decentralized or resource-limited settings, where rapid and comprehensive drug resistance testing remains a critical need.

This study has several limitations. First, due to the lack of phenotypic DST capability or failure to retain isolates in some participating centers, not all culture-positive patients underwent pDST or WGS, resulting in incomplete reference data. Second, the clinical impact of implementing the Sanity 2.0 assay as an initial diagnostic tool—particularly with regard to patient outcomes and cost-effectiveness—has yet to be evaluated. Third, PPA for INH and FQ resistance detection was slightly lower than that for RIF, which may be attributed to differences in probe design or mutation coverage between the Sanity 2.0 and comparator assays. Finally, due to limited specimen volume, discrepant and indeterminate samples were not subjected to repeat testing, which may have affected the final interpretation.

In summary, the Sanity 2.0 assay demonstrated robust diagnostic accuracy and operational advantages for the simultaneous detection of MTBC and resistance to RIF, INH, and FQ. Its rapid turnaround, ease of use, and compatibility with varied laboratory settings support its potential as a valuable tool for guiding timely clinical decisions, particularly in high-burden and resource-limited areas.

## Data Availability

All authors had full access to all the data in the study and take responsibility for the integrity of the data and the accuracy of the data analysis. Any supplementary materials cited in the article can be found in the online version.

## SUPPLEMENTAL MATERIAL

Supplemental tables jcm.01299-25-s0001.docx Tables S1 to S7.

ASM does not own the copyrights to Supplemental Material that may be linked to, or accessed through, an article. The authors have granted ASM a non-exclusive, world-wide license to publish the Supplemental Material files. Please contact the corresponding author directly for reuse.
