# Supplementary material for: Diagnostic performance of the Sanity 2.0 assay to detect resistance to rifampicin, isoniazid, and fluoroquinolones in tuberculosis
Source: J Clin Microbiol. 2025 Dec 10;64(1):e01299-25. doi: 10.1128/jcm.01299-25 (PMC12802206; doi:10.1128/jcm.01299-25)
Supplement: Supplemental tables — Tables S1 to S7. [file jcm.01299-25-s0001.docx]

**Table S1 Baseline characteristics of the patients**

| Variable | Overall  (n = 611) | MTBC-dectected  (n = 563) | MTBC-not dectected  (n = 48) | *P* value | |
| --- | --- | --- | --- | --- | --- |
| Age, median (IQR) | 41.0 (29.0, 53.0) | 41.0 (29.0,53.0) | 41.0 (27.0,54.0) | 0.618 | |
| Sex, n (%) |  |  |  | 0.770 | |
| Male | 423 (69.2%) | 391 (69.4%) | 32 (66.7%) |  | |
| Female | 185 (30.3%) | 169 (30.0%) | 16 (33.3%) |  | |
| Unknown | 3 (0.5%) | 3 (0.5%) | 0 (0.0%) |  | |
| Body mass index, median (IQR) | 20.0 (18.3, 22.2) | 20.0 (18.2,22.0) | 21.2 (19.1,24.2) | 0.014 | |
| Smoking history, n (%) |  |  |  | 0.004 | |
| Yes | 188 (30.8%) | 183 (32.5%) | 5 (10.4%) |  | |
| No | 381 (62.4%) | 344 (61.1%) | 37 (77.1%) |  | |
| Unknown | 42 (6.9%) | 36 (6.39%) | 6 (12.5%) |  | |
| Previous history of tuberculosis, n (%) |  |  |  | 0.060 | |
| Yes | 205 (33.6%) | 196 (34.8%) | 9 (18.8%) |  | |
| No | 364 (59.6%) | 331 (58.8%) | 33 (68.8%) |  | |
| Unknown | 42 (6.9%) | 36 (6.39%) | 6 (12.5%) |  | |
| Presence of cavity, n (%) |  |  |  | 0.035 | |
| Yes | 388 (63.5%) | 366 (65.0%) | 22 (45.8%) |  | |
| No | 181 (29.6%) | 161 (28.6%) | 20 (41.7%) |  | |
| Unknown | 42 (6.9%) | 36 (6.39%) | 6 (12.5%) |  | |
| Bilateral involvement, n (%) |  |  |  | 0.082 | |
| Yes | 435 (71.2%) | 408 (72.5%) | 27 (56.2%) |  | |
| No | 134 (21.9%) | 119 (21.1%) | 15 (31.2%) |  | |
| Unknown | 42 (6.9%) | 36 (6.39%) | 6 (12.5%) |  | |
| Smear, n (%) * |  |  |  | <0.001 | |
| Postive | 334 (54.7%) | 323 (57.4%) | 11 (22.9%) |  | |
| Negative | 229 (37.5%) | 199 (35.3%) | 30 (62.5%) |  | |
| Unknown | 48 (7.9%) | 41 (7.28%) | 7 (14.6%) |  | |
| Xpert MTB/RIF, n (%) |  |  |  | 1.000 | |
| Postive | 556 (91.0%) | 509 (90.4%) | 47 (97.9%) |  | |
| Negative | 2 (0.3%) | 2 (0.4%) | 0 (0.0%) |  | |
| Unknown | 53 (8.7%) | 52 (9.2%) | 1 (2.1%) |  | |
| Culture, n (%) |  |  |  | <0.001 | |
| Postive | 504 (82.5%) | 481 (85.4%) | 23 (47.9%) |  | |
| Negative | 55 (9.0%) | 42 (7.5%) | 13 (27.1%) |  | |
| Unknown | 52 (8.5%) | 40 (7.1%) | 12 (25.0%) |  |  |

Abbreviations: SD, standard deviation; IQR, interquartile range.

*Percentages may not sum to 100% due to rounding.

**Table S2 Diagnostic performance of the Sanity 2.0 assay for detection of drug resistance to RIF, INH, FQ compared with composite reference standard in BALF and Sputum.**

| **Drug** | **Reference standard** | **N** | **TP** | **FP** | **FN** | **TN** | **Sensitivity (% [95% CI])** | **Specificity (% [95% CI])** | **Accuracy (% [95% CI])** | **PPV (% [95% CI])** | **NPV (% [95% CI])** |
| --- | --- | --- | --- | --- | --- | --- | --- | --- | --- | --- | --- |
| BALF |  |  |  |  |  |  |  |  |  |  |  |
| RIF | pDST-NGS | 45 | 39 | 1 | 0 | 5 | 100.0 (91.0 - 100.0) | 83.3 (43.6 - 97.0) | 97.8 (88.4 - 99.6) | 97.5 (87.1 - 99.6) | 100.0 (56.6 - 100.0) |
| INH | pDST-NGS | 47 | 33 | 0 | 1 | 13 | 97.1 (85.1 - 99.5) | 100.0 (77.2 - 100.0) | 97.9 (88.9 - 99.6) | 100.0 (89.6 - 100.0) | 92.9 (68.5 - 98.7) |
| FQ | pDST-NGS | 30 | 13 | 2 | 0 | 15 | 100.0 (77.2 - 100.0) | 88.2 (65.7 - 96.7) | 93.3 (78.7 - 98.2) | 86.7 (62.1 - 96.3) | 100.0 (79.6 - 100.0) |
| Sputum |  |  |  |  |  |  |  |  |  |  |  |
| RIF | pDST-NGS | 271 | 204 | 2 | 1 | 64 | 99.5 (97.3 - 99.9) | 97.0 (89.6 - 99.2) | 98.9 (96.8 - 99.6) | 99.0 (96.5 - 99.7) | 98.5 (91.8 - 99.7) |
| INH | pDST-NGS | 256 | 156 | 0 | 11 | 89 | 93.4 (88.6 - 96.3) | 100.0 (95.9 - 100.0) | 95.7 (92.5 - 97.6) | 100.0 (97.6 - 100.0) | 89.0 (81.4 - 93.7) |
| FQ | pDST-NGS | 182 | 56 | 1 | 6 | 119 | 90.3 (80.5 - 95.5) | 99.2 (95.4 - 99.9) | 96.2 (92.3 - 98.1) | 98.2 (90.7 - 99.7) | 95.2 (89.9 - 97.8) |

Abbreviations: RIF, rifampicin; INH, isoniazid; FQ, fluoroquinolones; TP, true positive; FP, false positive; FN, false negative; TN, true negative; PPV, positive predictive value; NPV, negative predictive value; CI, confidence intervals.

**Table S3. Comparison of demographic and clinical characteristics between false-negative and true-positive cases in the isoniazid resistance analysis based on the composite reference.**

| Variable | Overall (n = 201) | FN (n = 12) | TP (n = 108) | *P* value |
| --- | --- | --- | --- | --- |
| Age, median (IQR) | 46.0 (33.0,55.0) | 52.0 (44.5,58.2) | 46.0 (33.0,54.5) | 0.133 |
| Sex, n (%) |  |  |  | 0.186 |
| Male | 145 (72.9%) | 11 (91.7%) | 134 (71.7%) |  |
| Female | 54 (27.1%) | 1 (8.3%) | 53 (28.3%) |  |
| Body mass index, median (IQR) | 20.1 (18.1,22.4) | 19.8 (18.9,23.2) | 20.1 (18.0,22.4) | 0.793 |
| Smoking history, n (%) |  |  |  | 1.000 |
| No | 114 (60.6%) | 6 (60.0%) | 108 (60.7%) |  |
| Yes | 74 (39.4%) | 4 (40.0%) | 70 (39.3%) |  |
| Previous history of tuberculosis, n (%) |  |  |  | 0.002 |
| No | 87 (46.3%) | 0 (0.0%) | 87 (48.9%) |  |
| Yes | 101 (53.7%) | 10 (100.0%) | 91 (51.1%) |  |
| Bilateral involvement, n (%) |  |  |  | 0.689 |
| No | 33 (17.6%) | 2 (20.0%) | 31 (17.4%) |  |
| Yes | 155 (82.4%) | 8 (80.0%) | 147 (82.6%) |  |
| Presence of cavity, n (%) |  |  |  | 0.125 |
| No | 39 (20.7%) | 0 (0.0%) | 39 (21.9%) |  |
| Yes | 149 (79.3%) | 10 (100.0%) | 139 (78.1%) |  |
| Smear, n (%) |  |  |  | 1.000 |
| Postive | 150 (80.2%) | 8 (80.0%) | 142 (80.2%) |  |
| Negative | 37 (19.8%) | 2 (20.0%) | 35 (19.8%) |  |
| Culture, n (%) |  |  |  | 0.176 |
| Postive | 190 (98.4%) | 11 (91.7%) | 179 (98.9%) |  |
| Negative | 3 (1.6%) | 1 (8.3%) | 2 (1.1%) |  |

Abbreviations: SD, standard deviation; IQR, interquartile range; FN, false negative; TP, true positive. Note: The total number may not equal the overall number because of missing or unknown data.

**Table S4.** **Comparison of demographic and clinical characteristics between Sanity-S/Xpert-R and Sanity-R/Xpert-R cases for fluoroquinolone resistance using Xpert MTB/XDR.**

| Variable | Overall (n = 92) | Sanity-S/Xpert-R (n = 11) | Sanity-R/Xpert-R (n = 81) | *P* value |
| --- | --- | --- | --- | --- |
| Age, median (IQR) | 43.5 (33.0,57.2) | 39.0 (29.0,57.0) | 45.0 (34.0,57.0) | 0.470 |
| Sex, n (%) |  |  |  | 0.450 |
| Male | 70 (76.1%) | 7 (63.6%) | 63 (77.8%) |  |
| Female | 22 (23.9%) | 4 (36.4%) | 18 (22.2%) |  |
| Body mass index, median (IQR) | 19.9 (18.2,22.1) | 21.1 (18.0,23.9) | 19.7 (18.3,21.2) | 0.615 |
| Smoking history, n (%) |  |  |  | 0.494 |
| No | 57 (68.7%) | 8 (80.0%) | 49 (67.1%) |  |
| Yes | 26 (31.3%) | 2 (20.0%) | 24 (32.9%) |  |
| Previous history of tuberculosis, n (%) |  |  |  | 0.329 |
| No | 37 (44.6%) | 6 (60.0%) | 31 (42.5%) |  |
| Yes | 46 (55.4%) | 4 (40.0%) | 42 (57.5%) |  |
| Bilateral involvement, n (%) |  |  |  | 1.000 |
| No | 15 (18.1%) | 2 (20.0%) | 13 (17.8%) |  |
| Yes | 68 (81.9%) | 8 (80.0%) | 60 (82.2%) |  |
| Presence of cavity, n (%) |  |  |  | 0.202 |
| No | 17 (20.5%) | 4 (40.0%) | 13 (17.8%) |  |
| Yes | 66 (79.5%) | 6 (60.0%) | 60 (82.2%) |  |
| Smear, n (%) |  |  |  | 0.212 |
| Postive | 65 (78.3%) | 6 (60.0%) | 59 (80.8%) |  |
| Negative | 18 (21.7%) | 4 (40.0%) | 14 (19.2%) |  |
| Culture, n (%) |  |  |  | 0.118 |
| Postive | 82 (94.3%) | 9 (81.8%) | 73 (96.1%) |  |
| Negative | 5 (5.7%) | 2 (18.2%) | 3 (3.9%) |  |

Abbreviations: SD, standard deviation; IQR, interquartile range; R, drug-resistant; S, drug- susceptible.

Note: The total number may not equal the overall number because of missing or unknown data.

**Table S5 Discordance in RIF resistance detection between the Sanity 2.0 assay and phenotypic drug susceptibility testing or whole-genome sequencing results**

| **Discordance** | **Participant ID** | **Sanity 2.0** | **pDST** | **WGS** | **WGS results** | **XPERT MTB/RIF** |
| --- | --- | --- | --- | --- | --- | --- |
| Discordance with pDST | 00773 | resistant | susceptible | resistant | *rpoB*_L430P | resistant |
|  | 00139 | resistant | susceptible | resistant | *rpoB*_L452P | resistant |
|  | 00361 | resistant | susceptible | resistant | *rpoB*_D435Y, *rpoB*_N437D | resistant |
|  | 00406 | resistant | susceptible | resistant | *rpoB*_S428R, *rpoB*_H445Q | resistant |
|  | 00679 | resistant | susceptible | resistant | *rpoB*_L430P | resistant |
|  | 00693 | resistant | susceptible | resistant | *rpoB*_L452P | resistant |
|  | 00334 | resistant | susceptible | resistant | *rpoB*_H445N | resistant |
|  | 00497 | resistant | susceptible | resistant | *rpoB*_L430P | resistant |
|  | 00603 | resistant | susceptible | resistant | *rpoB*_L430P | resistant |
|  | 00690 | resistant | susceptible | resistant | *rpoB*_L430P | resistant |
|  | 00177 | resistant | susceptible | resistant | *rpoB*_D435V | resistant |
|  | 00481 | resistant | susceptible | resistant | *rpoB*_H445D | resistant |
|  | 00153 | resistant | susceptible | resistant | *rpoB*_H445L | resistant |
|  | 00561 | resistant | susceptible | ND | ND | resistant |
|  | 00604 | resistant | susceptible | ND | ND | resistant |
|  | 00671 | resistant | susceptible | ND | ND | resistant |
| Discordance with WGS | 00444 | resistant | susceptible | susceptible | WT | resistant |
|  | 00606 | resistant | ND | susceptible | *rpoB*_L957V | resistant |
|  | 00155 | resistant | ND | susceptible | WT | resistant |
|  | 00398 | susceptible | susceptible | resistant | *rpoB*_V170F | ND |
| Discordance with pDST and WGS | 070009 | resistant | susceptible | susceptible | WT | susceptible |
|  | 070011 | resistant | susceptible | susceptible | WT | susceptible |

Abbreviations: RIF, rifampicin; pDST, phenotypic drug susceptibility testing; WGS, whole-genome sequencing; ND, not done.

**Table S6 Discordance in INH resistance detection between the Sanity 2.0 assay and phenotypic drug susceptibility testing or whole-genome sequencing results**

| **Discordance** | **Participant ID** | **Sanity 2.0** | **pDST** | **WGS** | **WGS results** | **XPERT MTB/XDR** |
| --- | --- | --- | --- | --- | --- | --- |
| Discordance with pDST | 00460 | resistant | susceptible | resistant | *katG*_S315T | resistant |
|  | 00636 | resistant | susceptible | resistant | *fabG1*_c-15t | resistant |
|  | 00548 | resistant | susceptible | resistant | *ahpC*_g-74a | susceptible |
|  | 00398 | resistant | susceptible | resistant | *katG*_S315T | resistant |
|  | 00177 | resistant | susceptible | resistant | *katG*_S315T | resistant |
|  | 00288 | resistant | susceptible | ND | ND | susceptible |
|  | 00039 | susceptible | resistant | susceptible | *katG*_G299S | resistant |
|  | 00081 | susceptible | resistant | susceptible | *katG*_L48P | resistant |
|  | 00615 | susceptible | resistant | susceptible | *katG*_N660D, *katG*_G169A | resistant |
|  | 00266 | susceptible | resistant | susceptible | *katG*_Y229C | susceptible |
|  | 00531 | susceptible | resistant | susceptible | *katG*_L430P | susceptible |
|  | 00618 | susceptible | resistant | susceptible | *katG*_W300G | susceptible |
|  | 00412 | susceptible | resistant | ND | ND | susceptible |
|  | 00308 | susceptible | resistant | ND | ND | resistant |
|  | 00475 | resistant | resistant | susceptible | *ahpC*_g-48a | resistant |
|  | 00444 | resistant | resistant | susceptible | WT | resistant |
| Discordance with WGS | 00481 | resistant | resistant | susceptible | *ahpC*_c-57t, *katG*_A291V | resistant |
|  | 00711 | resistant | ND | susceptible | *ahpC*_g-48a, *katG*_D163E | resistant |
|  | 00630 | resistant | ND | susceptible | *ahpC*_c-57t | resistant |
|  | 00453 | resistant | ND | susceptible | *katG*_D142A, *ahpC*_g-74a | susceptible |
|  | 00347 | susceptible | ND | resistant | *katG*_152A_deletion | resistant |
|  | 00426 | susceptible | ND | resistant | *katG*_W198* | susceptible |
| Discordance with pDST and WGS | 00449 | susceptible | resistant | resistant | *katG*_S315T | susceptible |
|  | 00459 | susceptible | resistant | resistant | *katG_*S315T | susceptible |

Abbreviations: INH, isoniazid; pDST, phenotypic drug susceptibility testing; WGS, whole-genome sequencing; ND, not done.

**Table S7 Discordance in FQ resistance detection between the Sanity 2.0 assay and phenotypic drug susceptibility testing or whole-genome sequencing results**

| **Discordance** | **Participant ID** | **Sanity 2.0** | **pDST** | **WGS** | **WGS results** | **XPERT MTB/XDR** |
| --- | --- | --- | --- | --- | --- | --- |
| Discordance with pDST | 00336 | resistant | susceptible | resistant | *gyrA*_A90V | resistant |
|  | 00365 | resistant | susceptible | resistant | *gyrA*_D94G | resistant |
|  | 00148 | resistant | susceptible | resistant | *gyrA*_D94G | resistant |
|  | 00523 | resistant | susceptible | resistant | *gyrA*_A90V | resistant |
|  | 00683 | resistant | susceptible | ND | ND | resistant |
|  | 00520 | resistant | susceptible | ND | ND | susceptible |
|  | 040054 | resistant | susceptible | ND | ND | susceptible |
|  | 00368 | resistant | susceptible | ND | ND | ND |
|  | 00496 | susceptible | resistant | susceptible | *gyrA*_S779P | susceptible |
|  | 00475 | susceptible | resistant | susceptible | WT | susceptible |
|  | 00459 | susceptible | resistant | susceptible | *gyrA*_P8H | resistant |
| Discordance with WGS | 00449 | resistant | resistant | susceptible | WT | resistant |
|  | 00314 | resistant | ND | susceptible | WT | resistant |
|  | 00152 | resistant | ND | susceptible | WT | resistant |
|  | 00305 | resistant | ND | susceptible | WT | resistant |
|  | 00150 | resistant | susceptible | susceptible | *gyrA*_D89N | resistant |
|  | 00382 | resistant | ND | susceptible | *gyrA*_D94V | susceptible |
|  | 00639 | susceptible | ND | resistant | *gyrA*_A90V | susceptible |
|  | 00015 | susceptible | ND | resistant | *gyrA*_D94G | susceptible |
|  | 00290 | susceptible | ND | resistant | *gyrB*_D461N | susceptible |
| Discordance with pDST and WGS | 00442 | resistant | susceptible | susceptible | WT | susceptible |
|  | 00444 | resistant | susceptible | susceptible | WT | susceptible |

Abbreviations: FQ, fluoroquinolones; pDST, phenotypic drug susceptibility testing; WGS, whole-genome sequencing; ND, not done.
